# “How involved do you feel?” The PILS-Stroke questionnaire: a Rasch-built measure of social participation after stroke

**DOI:** 10.3389/fneur.2026.1733609

**Published:** 2026-05-11

**Authors:** Edouard Ducoffre, Yannick Bleyenheuft, Massimo Penta, Merlin Somville, Zélie Rosselli, Geoffroy Saussez, Yves Vandermeeren, Carlyne Arnould

**Affiliations:** 1 Motor Skill Learning and Intensive Neurorehabilitation Lab, Institute of Neuroscience, UCLouvain, Louvain-la-Neuve, Belgium; 2 Forme & fonctionnement Humain (FfH) Unit, CeREF-Santé, Haute Ecole Louvain en Hainaut, Montignies-sur-Sambre, Belgium; 3 Arsalis SRL, Glabais, Belgium; 4 Institute of Neuroscience, UCLouvain, Louvain-la-Neuve, Belgium; 5 Stroke Unit/Motor Learning Lab, Neurology Department, CHU UCL Namur, site Godinne, UCLouvain, Yvoir, Belgium; 6 NEUR Department, Institute of Neuroscience, UCLouvain, Brussels, Belgium

**Keywords:** International Classification of Functioning, Disability and Health (ICF), Involvement, Neurorehabilitation, Patient-reported outcome measure (PROM), Rasch, Social Participation, Stroke

## Abstract

**Introduction:**

Advances in acute stroke management have increased the number of individuals living with long-term disabilities, presenting challenges in maintaining prior levels of participation in life situations. Return to active participation can be seen as the goal of rehabilitation, given its clear impact on patients‘ quality of life. In this study, we aimed to develop the Participation in Life Situations-Stroke (PILS-Stroke) questionnaire, a self-reported Rasch-built tool for measuring patients' social involvement in meaningful life situations.

**Methods:**

We assembled a 72-item experimental version of PILS-Stroke, which was grounded on patients' and experts' perspectives via an initial item content review followed by item relevance/comprehensibility assessment. We then administered the questionnaire to 105 post-stroke individuals (58% males; mean ± SD: 62 ± 14 years) discharged for at least one month from hospital. Participants rated their involvement in life situations using a 3-point scale (0: “I would like to, but I don't get involved”; 1: “I get involved a little”; 2: “I get involved a lot”; ?: “I don't know/I don't want to get involved”). The responses were analyzed using the Rasch measurement model (RUMM2030+ software) to select the items presenting the best psychometric properties, resulting in an objective and unidimensional measurement tool. Construct validity was assessed using ten clinical measures covering International Classification of Functioning, Disability, and Health (ICF) domains (body functions, activities, participation).

**Results:**

The final 38-item PILS-Stroke demonstrated good reliability [Person Separation Index (PSI) = 0.89] and defined a unidimensional and linear scale for measuring stroke patients' social participation. There was a high correlation between social participation with satisfaction regarding activities/participation (SATIS-Stroke, r_s_ = 0.7, *P* < 0.001) and weak-to-moderate correlation with performance of motor activities (ACTIVLIM-CS, ABILHAND-CS, ABILOCO-CS; 0.20 ≤ r_s_ ≤ 0.39, *P* < 0.049) and certain psychological indicators (depression [HADS], r_s_ = −0.45, *P* < 0.001; confidence [CaSM], r_s_ = 0.47, *P* < 0.001).

**Conclusions:**

PILS-Stroke is a valid and reliable unidimensional tool specifically developed to measure stroke patients' social involvement in life situations. Its psychometric properties show promising potential for monitoring patients' social participation and quantifying the effectiveness of rehabilitation programs promoting their social inclusion.

## 1 Introduction

Stroke represents the third-leading cause of death and disability in adults, with current estimates suggesting a lifetime 25% risk of stroke (1, 2). Improvements in acute management have resulted in growing numbers of stroke survivors, many of whom suffer from long-term disabilities (2). A transition to long-term disability presents stroke survivors with challenges in maintaining their previous level of social participation (3). Additionally, the growing incidence of stroke in younger populations (4) significantly restricts their social involvement in critical life domains such as employment and productivity (5) and may thus have a substantial detrimental effect on their quality of life (6).

Participation, as defined in the International Classification of Functioning, Disability, and Health (ICF), refers to “involvement in life situations” (7). However, the lacking precision of this definition has contributed to conceptual overlap and confusion with certain related constructs, including daily activities (8). Research has been conducted to clarify the scope of social participation, highlighting its essential components such as the distinction between attendance and involvement, interactions with others and social roles, and preferences of the individual related to their interests and the value placed on life situations (8–10). In addition, social participation is shaped by a range of barriers and facilitators, including personal and environmental factors, highlighting the highly individualized nature of this concept (8).

Considering these key components, we propose a more comprehensive definition of social participation as follows: the involvement of people in life situations, chosen by individuals because they make sense to them, in which they may interact with others, or which relate to social roles. Involvement is thus not limited to witnessing a life situation, but entails feeling that one is experiencing that situation and devoting one's attention to it. This is manifest through social interactions and with a certain degree of motivation and commitment, e.g., being attentive, giving one's opinion, encouraging, performing tasks, or helping others to perform them.

Post-stroke rehabilitation should target maximizing the survivor's autonomy and participation in daily life situations (11) as these appear to be strong determinants of quality of life (6). Assessing the effects of interventions extending beyond performance of motor functions and completion of activities is therefore essential to get a full understanding of a patient's functioning and specific needs. Measuring their social participation would help to identify the most restricting life situations faced by stroke patients, thereby informing the tailored development of targeted and adapted interventions that would best facilitate social reintegration and support continuity of life after stroke.

Although there is an increasing number of studies prioritizing participation as a primary outcome, its assessment in routine clinical practice remains limited due to the unavailability of adequate, valid, and clinically meaningful assessment tools (12). The participation measurement tools that are available present several disadvantages. First, most of are generic; paying little heed to specific aspects of the intended medical condition, they are consequently insufficiently sensitive to clinical changes (13) (e.g., LIFE-H (14), and Frenchay Activities Index (15)). Second, they often include items related to daily activities which do not reflect the social interactions or roles that underlie the concept of participation, making their clinical interpretation more complex (e.g., SATIS-Stroke (16), and Stroke Impact Scale-Participation (17)). Third, most measurement tools are focused on patients' attendance (i.e., number of activities/situations performed or number of times the activity is performed) rather than on patients' involvement, a criterion that is obviously necessary, but not sufficient for patient's participation. (18) Such tools do not take into account patients' preferences, for example by failing to confine consideration only to those life situations that make sense to an individual (e.g., LIFE-H (14), Frenchay Activities Index (15), and Stroke Impact Scale-Participation (17)). Finally, previous research has emphasized the inadequate psychometric qualities (e.g., linearity, unidimensionality, construct validity, and test-retest reliability) of some participation measures, which limits their clinical interpretability (19).

In response to these observations, we aimed in this study to develop and validate the PILS-Stroke questionnaire, a new Patient-Reported Outcome Measure (PROM) measuring exclusively social participation in the specific population of post-stroke adults through their perceived involvement in life situations that make sense to them. The scale was developed using the Rasch model, a probabilistic model increasingly employed in the refinement of health measurement tools. The Rasch model converts ordinal data into linear measures, enabling quantitative comparisons across individuals and time (20). Moreover, we used the model to verify that the scale meets the requirements of an objective measurement through selection of those items presenting the best psychometric qualities.

## 2 Materials and methods

This study was conducted by the Motor Skill Learning and Intensive Neurorehabilitation Laboratory (MSL-IN Lab, Institute of Neuroscience (IoNS), UCLouvain), in collaboration with the HELHa, in Brussels, Belgium. Ethical approval was obtained from the Ethics Committee of Saint-Luc – UCLouvain, Belgium (clinical trial number: B4032022000142). Assessments were conducted either at the participants' home or at the IoNS (Brussels, Belgium).

### 2.1 Participants

We targeted a sample size of one hundred participants. This sample size is generally regarded as adequate for scale development and evaluation using Rasch analysis in health outcomes research (21). Participants were recruited via email or phone calls using pre-existing contact lists, word-of-mouth referrals, or through outreach at the “*Center Hospitalier Universitaire (CHU) UCL Namur*” in Belgium.

### 2.2 Eligibility criteria

Eligible participants were aged between 18 and 90 years, had a confirmed diagnosis of ischemic or hemorrhagic stroke, had been discharged from hospital at least 1 month prior to assessment, and were able to provide informed consent. Exclusion criteria included a diagnosis of transient ischemic attack (TIA) instead of stroke, residence in a facility without access to cultural or leisure group activities and a visitors' social room, temporary health conditions limiting autonomy (e.g., acute orthopedic issues or illness), diagnosis of another chronic neurological disorder, severe cognitive impairment (MoCA/MMSE score < 10/30 at the time of assessment), or refusal to participate in the evaluation.

### 2.3 Instrument

Item selection was based on preexisting measurement tools and screening, including an initial item content review by three physical therapy researchers followed by an item relevance/comprehensibility assessment conducted by five chronic stroke survivors and five physiotherapists. This procedure led to formulation of the 72-item experimental PILS-Stroke questionnaire (see Supplementary Material). This questionnaire was presented to participants in ten different random orders, to avoid a potential order bias. Each item in the questionnaire describes a life situation in which participants were asked to evaluate their level of social involvement using a three-level ordinal scale: “I would like to, but I don't get involved” (0), “I get slightly involved” (1), and “I get very involved” (2). An additional response option, “I don't know / I don't want to participate” (?), was available for situations deemed by the participant as irrelevant or lacking in personal interest. This latter option was treated as a missing value in the Rasch model analysis, as the instrument is intended to capture social involvement only in meaningful life situations. More details about PILS-Stroke instructions are provided in Supplementary Material.

### 2.4 Procedures

First, after giving their written, informed consent, eligible patients were asked to complete a general information questionnaire including various sociodemographic (i.e., age, sex) and clinical information (i.e., more affected side, time since stroke, stroke type). Second, the Montreal Cognitive Assessment (MoCA) (22) or the Mini-Mental State Examination (MMSE) (23) were used to evaluate participants' cognitive state. Both scales range from 0 to 30, with scores of 27–30 indicating intact cognitive state, and scores below 10 indicated a severe cognitive deficit. We thus ensured exclusion of subjects with major cognitive impairment.

We next administered several clinical assessments. The first three evaluations (i.e., modified Rankin Scale, and pain and fatigue visual analog scales) were consistently conducted just prior to completion of the PILS-stroke questionnaire. The modified Rankin Scale (mRS) (24) classifies stroke survivors according to their degree of disability on a 6-level scale (i.e., from 0 “no symptoms” to 5 “severe disability”). Pain and fatigue were assessed though visual analog scales (VAS) extending from 0 (absent) to 10 (severe). Afterwards, the participants completed the 72 item PILS-Stroke questionnaire, followed by completion of eight clinical assessments (presented in random order). These additional assessments (*vide infra*) as well as pain and fatigue VAS were used to examine the convergent construct validity of the PILS-Stroke scale by analyzing its relationship with factors theoretically related to study social participation.

Among the eight clinical assessments, we measured anxiety and depression using the Hospital Anxiety and Depression Scale (HADS) (25), which is a 14-item questionnaire comprising subscales for depressive (HADS-D; 7 items) and anxious (HADS-A; 7 items) symptoms. Each item is scored from 0 (absent) to 3 (severe), leading to a maximum score of 21 for each subscale. Post-stroke confidence was evaluated with the Confidence after Stroke Measure (CaSM) (26), a 27-item scale covering self-confidence (9 items), positive attitude (8 items), and social confidence (10 items) subdomains on a four-level scale extending from 0 (absent) to 3 (pronounced), giving a maximum total score of 81, with higher values indicating greater confidence.

The Timed Up and Go (TUG) (27) test of the time (in seconds) to stand up from a chair, walk three meters, turn around, and return to sitting position, was performed to assess functional mobility. We also applied three Rasch-built patient-reported outcome measures, namely ABILOCO-CS (28) (13 items), ABILHAND-CS (29) (23 items), and ACTIVLIM-Stroke (30) (20 items), to assess locomotor, bimanual, and global activity performance, respectively, each on a linear 0–100 scale, with higher scores indicating better performances.

Satisfaction regarding activities and participation was evaluated using the Rasch-built SATIS-Stroke scale (16) (0–100 units), with higher scores indicating greater satisfaction. Finally, health-related quality of life was assessed with the EuroQol-5 Dimensions-5 Levels (EQ-5D-5L) (31), generating a five-domain health profile that is converted into a utility index score using the Belgian normative value set ranging from −0.532 to 1 units, with higher values indicating better quality of life.

Most of these measurements qualify as patient-reported outcome measures (PROMs); however, some participants faced challenges in answering questionnaires autonomously due to their post-stroke deficits (e.g., visual impairment, unilateral neglect, aphasia, or apraxia) and consequently requested varying levels of assistance from the examiner. Additionally, some participants preferred to have their answers transcribed by the examiner. The examiner also answered basic questions regarding item comprehension. If an item remained unclear, participants were instructed to skip it, which would be recorded as a missing value for the Rasch-built questionnaires.

### 2.5 Data analysis

#### 2.5.1 Rasch analysis

Participants' responses to the PILS-Stroke experimental questionnaire were analyzed using the Rasch model RUMM2030+ software (32) (RUMM Laboratory Pty; Ltd., Perth, Western Australia). The Rasch model estimates the patients' ability, item difficulty and item thresholds (i.e., the social participation level for which two successive response categories have the same probability of occurrence) by integrating responses on an ordinal scale (33) within a probabilistic framework to build an interval scale and obtain linear measures (20). These linear measures are expressed in logits, which is a constant measurement unit that is reproducible across the entire measurement scale. To provide more readily comprehensive measures, we transformed the logit unit into a 0–100 PILS unit range, 0 PILS units represents the lowest social involvement and 100 PILS units the highest.

In this study, we preferred the Rating scale model (RSM) over the partial credit model (PCM), implying that the distance between threshold locations (i.e., corresponding to the middle response category “I get slightly involved”) is constrained to be similar across all items. Only items presenting a response rate ≥ 50% were retained, as they make sense to the majority of stroke patients in our sample.

Finally, successive Rasch analyses were used to ensure that retained items met the criteria for an objective measurement (see Detailed Methods). More specifically, only relevant items presenting the following criteria were retained: (1) **well-discriminated response categories**, including ordered responses categories (i.e., ensuring that higher social involvement levels correspond to higher response choices) and similar distance between thresholds locations (i.e., ensuring the RSM use); (2) **similar relative thresholds** in order to apply the RSM; (3) **unidimensionality** (i.e., adequate fit between observed and predicted responses); (4) **invariant item difficulty hierarchy (i.e., differential item functioning [DIF])** across age ( ≤ 63 years [median age] vs. > 63 years), sex, disability level, time since stroke ( ≤ 37 months [median time] vs. > 37 months), and risk of anxiety/depression (Hospital Anxiety and Depression Scale (HADS) ≤ 12 [median score] vs. > 12); and (5) **local independence**, ensuring that responses to one item are independent from the responses to another.

#### 2.5.2 Targeting and reliability

Item-patient targeting was investigated by comparing patients' group mean measure to the scale's mean difficulty (50 PILS units) to verify whether the scale difficulty was adapted for the sample. Floor and ceiling effects were considered non-substantial when < 15% of the patients achieved the minimum (0 PILS units) or maximum (100 PILS units) score on the scale (34). The scale reliability in our sample was evaluated using the Rasch Person Separation Index (PSI), calculated as the ratio of true to observed measure variances. This index helps to determine the number of distinct social involvement levels that can be statistically differentiated within the sample (35).

#### 2.5.3 Construct validity

Convergent construct validity was investigated through the relationships between PILS-Stroke measures and patients' sociodemographic (i.e., age, sex) and clinical factors (i.e., stroke type, time since stroke, degree of disability), as well as clinical measures assessing patients' body functions, activities, activities and participation, and health-related quality of life (see Procedures). The hypothesized associations between PILS-Stroke social participation measures and other constructs were grounded in the Family of Participation-Related Constructs framework and findings in the previous literature (6, 9, 36, 37).

We hypothesized that PILS-Stroke measures would be significantly associated with disability level and most clinical assessments of body functions, activities, participation, and quality of life, but not with sex, stroke type, nor time since stroke. Relationships with age were expected, but considered less certain, as disability may mask their impact on social participation. We used nonparametric statistics to investigate the relationships between PILS-stroke measures and other constructs, as the data did not meet normality and/or homoscedasticity assumptions. Thus, Spearman correlations were computed for continuous or pseudo-continuous variables (i.e., age, time since stroke, VAS, PROMs), Mann-Whitney tests for two-group nominal variables (i.e., sex and stroke type), and Kruskal-Wallis ANOVA on ranks for more than two groups (i.e., degree of disability). The significance threshold was set at p < 0.05, with statistical analyses performed in SigmaPlot 14.5.

## 3 Results

### 3.1 Participants

A total of 105 participants (58% males; mean ± SD: 62 ± 14 years) were recruited to develop the PILS-Stroke scale. Most participants had experienced an ischemic stroke at least 1 year before the study. Despite the persistence of functional difficulties such as balance disorders or apraxia, eight participants did not report a clearly affected side. All five levels of the modified Rankin Scale (mRS) were observed in our sample, with most reporting levels 1 to 3. Demographic and clinical characteristics of the sample are presented in Table 1.

**Table 1:** Sample description (*n*=105).

**Age**, years, mean ± SD (range)	61.8 ± 14.3 (26–97)
Sex, *n* (%)	
Male	61 (58.1%)
Female	44 (41.9%)
More affected side, *n* (%)	
Right	41 (39.0%)
Left	55 (52.4%)
None	8 (7.6%)
Stroke type, *n* (%)	
Ischemic	69 (65.7%)
Hemorrhagic	31 (29.5%)
Missing data	5 (4.8%)
Time since stroke, months, [Q1–Q3] (range)	37 [17.5–79.5] (2–504)
Degree of disability, *n* (%)	
mRS0	4 (3.8%)
mRS1	25 (23.8%)
mRS2	30 (28.6%)
mRS3	41 (39%)
mRS4	2 (1.9%)
mRS5	3 (2.8%)

SD, standard deviation; n, number; mRS, modified Rankin Scale; Q, quartiles.

### 3.2 Item selection of PILS-Stroke

One item was removed because the response rate was less than 50%, indicating that this life situation was not relevant for most stroke patients in the sample. The three-level response scale of 9 items were not well discriminated by participants, and 4 items did not share a similar rating scale. These 13 items were therefore removed, and a RSM was then applied. In addition, two items were discarded because their responses were dependent on the responses to other items. Four items failed to conform to the unidimensionality requirement and 14 additional items presented differential item functioning (DIF) as their difficulties varied across sex (*n* = 4), age (*n* = 6), degree of disability (*n* = 4), time since stroke (*n* = 2), and risk of anxiety/depression (*n* = 2), with several items showing DIF across multiple characteristics (*n* = 4). These items were removed along with misfitting items. One remaining item continued to exhibit a marginally significant DIF across sex (i.e., “Call someone I don't know by phone or cell phone”), and was retained due to its clinical relevance and good targeting within the sample. Details about item selection are available in Supplementary Material.

### 3.3 Description of PILS-Stroke

The calibration of the 38 PILS-Stroke items is presented in Table 2. The easiest items are mainly related to interactions between individuals that are possible without physical engagement (e.g., “Communicate with people around me”), while most of the more difficult items refer to situations that are physically demanding, especially moving around (e.g. “Use public transportation”). The scale covers all different dimensions of participation domain in the ICF (7): d1 learning and knowledge (4 items), d2 general tasks (5 items), d3 communication (8 items), d4 mobility (5 items), d5 self-care (2 items), d6 domestic life (6 items), d7 interpersonal interactions (12 items), d8 major life areas (3 items) and d9 community, social & civic life (9 items), with 16 items belonging to multiple subdomains. The scale presents a non-significant χ^2^ for item-trait interaction indicating that, overall, the 38 items contribute to the definition of a unidimensional social participation measure. This is confirmed by individual item fit statistics shown in Table 2.

**Table 2:** Calibration of the 38 PILS-Stroke items.

Item		Item difficulty (PILS unit)	SE (PILS unit)	FitResid (z)	Chi ^2^ (*p*-value)
1	Fulfill my role as a parent ^d6, d7^	41.9	2.2	−0.91	0.07
2	Communicate with people around me ^d3^	42.4	2	−1.17	0.37
3	Communicate with a loved one by text or on social media ^d3^	43.2	2.1	0.29	0.19
4	Request help in case of emergency ^d3^	43.5	2	1.73	0.16
5	Call someone I know by phone or cell phone ^d3^	43.7	1.9	0.58	0.12
6	Manage how and with whom I spend my time ^d2^	44.7	1.9	−0.44	0.07
7	Maintain ties with my siblings ^d7^	45.1	2.2	0.25	0.13
8	Perceive the emotions of others (happy, angry, worried, surprised...) ^d1, d7^	45.4	1.9	−0.08	0.28
9	Manage my money ^d8^	45.4	1.9	0.13	0.27
10	Support my loved ones in their projects ^d7^	45.5	1.9	−1.29	0.06
11	Get involved in a couple's relationship ^d7^	46	2.1	−0.01	0.29
12	Stand up for my rights ^d8, d9^	46	1.9	−0.65	0.82
13	Choose activities based on my priorities ^d1, d2^	46.4	1.8	0.48	0.15
14	Go out to a restaurant ^d9^	46.4	1.8	1.35	0.52
15	Express my desires to my loved ones ^d3, d7^	48.1	1.8	0.47	0.21
16	Help with preparing to host friends/family at my home ^d6^	48.5	1.9	−1.05	0.51
17	Go eat at a friend's house ^d7, d9^	48.7	1.8	−1.19	0.23
18	Participate in the preparation of simple meals (sandwiches, toast, snacks...) ^d6^	49.4	1.9	1.26	0.18
19	Maintain a pleasant environment that suits everyone at home (tidiness, cleanliness, organization...) ^d6^	49.7	1.7	−0.87	0.71
20	Plan ahead to make it easier to do activities with other people ^d2^	49.8	1.9	−0.86	0.31
21	Access outdoor spaces at/around my home to accompany my loved ones ^d4^	49.9	1.7	−0.34	0.95
22	Visit a friend ^d7, d9^	50.6	1.7	−1.89	0.09
23	Express disagreement politely ^d3, d7^	50.9	1.7	0.23	0.86
24	Get out of the house to do the activities I choose ^d2, d4^	51.2	1.7	1.31	0.72
25	Move around the community ^d4^	51.2	1.8	−0.95	0.15
26	Debate with someone by putting forward arguments ^d1, d3^	52.2	1.8	−0.21	0.09
27	Maintain relationships with colleagues (work, volunteering, clubs...) ^d7, d8^	52.6	1.8	−0.24	0.23
28	Deal with issues in the community the moment they arise (unexpected events, conflicts...) ^d2, d9^	53.2	1.7	−1.38	0.87
29	Supervise children playing ^d6, d7^	53.4	2.1	1.42	0.88
30	Enjoy a hobby (gardening, DIY, board games, card games, knitting...) ^d9^	53.6	1.7	0.38	0.39
31	Call someone I don't know by phone or cell phone ^d3^	53.9	1.9	0.5	0.15
32	Feel fulfilled in my sexual life ^d5, d7^	55	2	−0.63	0.45
33	Help prepare more elaborate meals like a starter, main dish, or dessert ^d6^	55.2	1.8	−1.1	0.7
34	Do recreational physical activity (walks, yard games...) ^d9^	55.4	1.7	1.46	0.6
35	Try on new clothes in stores ^d5, d9^	58	1.9	−0.55	0.22
36	Use public transportation ^d4^	59.7	2.3	1.77	0.61
37	Take walks with friends or family on uneven terrain (forest, beach...) ^d4^	59.7	1.8	0.92	0.16
38	Organize a party ^d1, d9^	61.4	1.9	−0.35	0.87

Items are sorted by increasing difficulty from top to bottom. Higher PILS units (range: 0–100) indicate more difficult situations. SE: standard error. ICF participation subdomains: d1: Learning and applying knowledge, d2: General tasks and demands, d3: Communication, d4: Mobility, d5: Self-care, d6: Domestic life, d7: Interpersonal interactions and relationships, d8: Major life areas, d9: Community, social and civic life.

**Figure 1:**
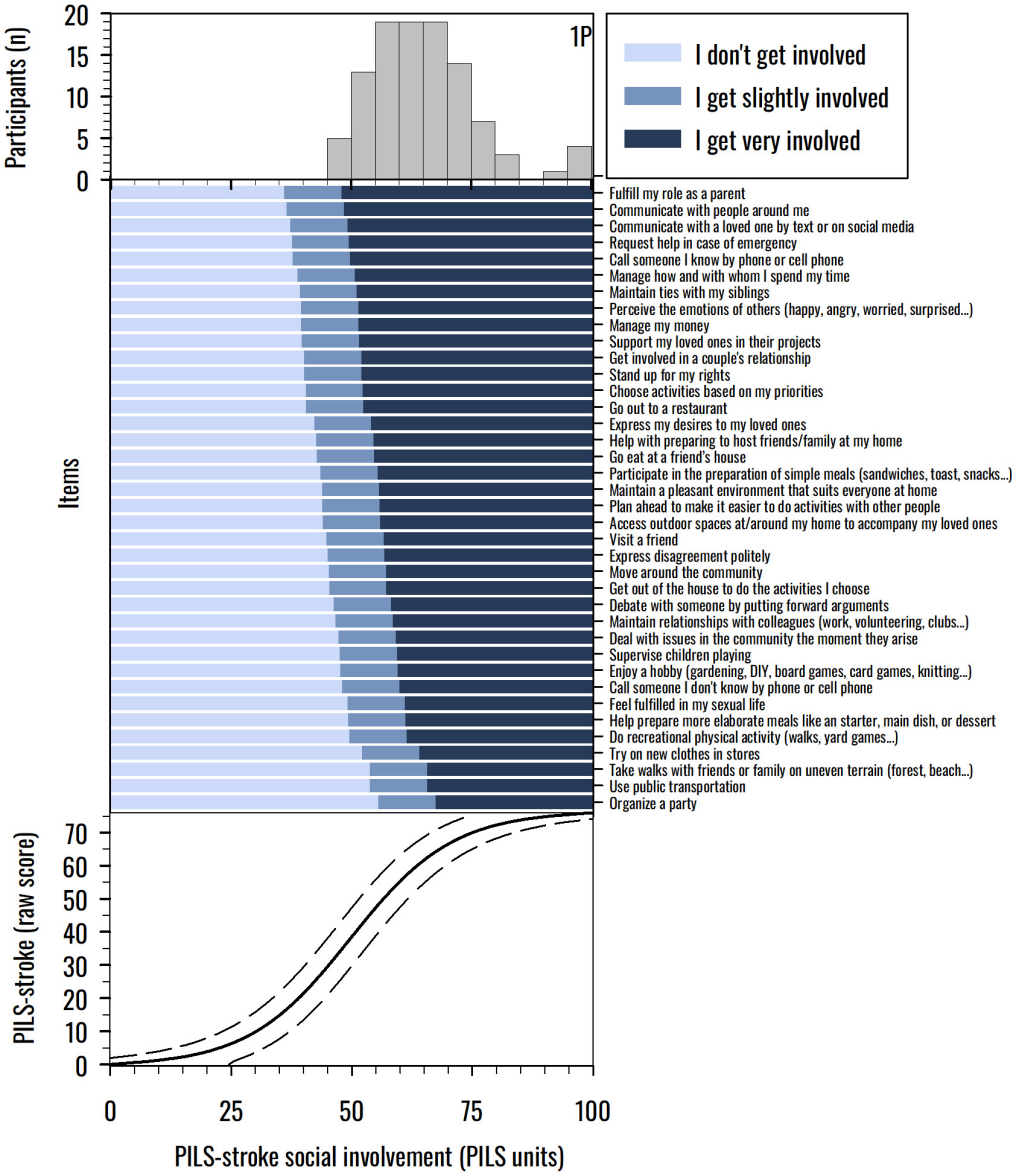
PILS-Stroke item map. Top panel: distribution of PILS-Stroke measures (expressed in PILS units) of stroke survivors (*n* = 105, one participant (1P) with a maximal score (100 PILS units) could not be measured by the scale because he was involved in all life situations). Middle panel: individuals' most probable response (“I would like to, but I don't get involved” in light blue, “I get slightly involved” in medium blue, and “I get very involved” in dark blue) to each item as a function of the underlying social participation measurement. The 38 PILS-Stroke items are sorted from top to bottom by increasing difficulty. Bottom panel: sigmoidal curve showing the relationship between ordinal total raw scores (from 0 to 76) and linear PILS-Stroke measures expressed in PILS units (solid line) and its 95% confidence interval considering the standard errors (dotted lines).

PILS-Stroke is depicted in Figure 1. The patients' mean social involvement measure is equal to 60.4 PILS units (range: 40.5–100), while the mean item difficulty is by convention set to 50 PILS units (threshold range: 36–67.3), indicating that the scale is particularly easy for our sample to complete, or that our sample performed particularly well for the scale difficulty (top and middle panels of Figure 1). However, only one participant achieved the maximum score of 100 PILS units, and none obtained the minimum score of 0 PILS unit, highlighting the absence of floor and ceiling effects. The PSI was equal to 0.89, indicating that the scale has a good internal consistency, enabling four levels of social involvement to be statistically distinguished in our sample. The middle panel of Figure 1 shows the most probable response to a given item as a function of the underlying PILS-Stroke measure. By comparing the social involvement of a given stroke patient to the difficulty of each item, it is possible to determine the patients' most probable scores to the items. The bottom panel of Figure 1 illustrates the nonlinear relationship between ordinal total raw scores and PILS-Stroke measures.

### 3.4 Construct validity

**Figure 2:**
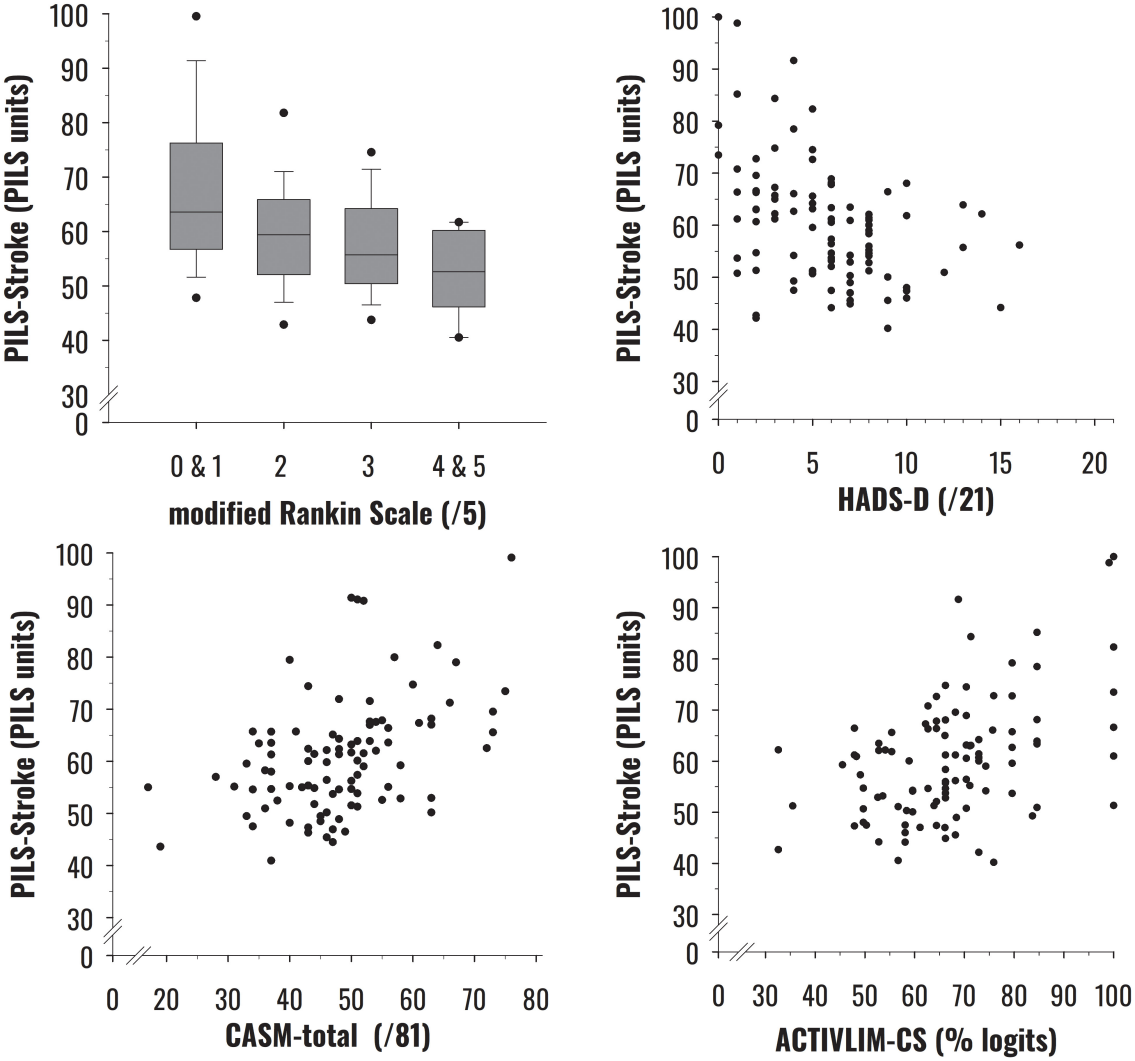
Relationships between social involvement in life situations (PILS-Stroke) and four clinical indicators: degree of disability (modified Rankin Scale [mRS]); depressive symptoms (depressive subscale of the Hospital Anxiety and Depression Scale [HADS-D]); Confidence after a stroke [total score of the Confidence after Stroke Measure (CASM-total); and global performance in daily activities (ACTIVLIM-CS)].

Convergent construct validity results are summarized in Table 3 and illustrated in Figure 2. See Supplementary Material for the description of participants' clinical measures. As hypothesized, PILS-Stroke measures were significantly associated with the degree of disability and most clinical assessments testing body functions, activities, activities & participation, and quality of life. The strongest association was observed with satisfaction regarding activities and participation (SATIS-Stroke), while moderate relationships were found with depression, global confidence, and self-confidence measures. Weaker yet still significant correlations were also observed with locomotor and global activity performances, and, to a lesser extent, with fatigue, positive attitude and social confidence, functional mobility, manual ability, and health-related quality of life.

**Table 3:** Relationships between PILS-Stroke measures and patients' sociodemographic & clinical factors, and clinical measures (body functions, activities, activities & participation, and quality of life).

Variable	Assessment tool	Statistical test	*p*-value
Sociodemographic factors			
Age		r_s_ = −0.19	0.053
Sex		U = 1169	0.261
Clinical factors			
Stroke type (ischemic/hemorrhagic)		U = 836.5	0.083
Time since stroke		r_s_ = 0.01	0.913
Degree of disability	mRS	H = 9.57	0.023
**ICF domain: body functions**			
Pain	VAS pain	r_s_ = −0.04	0.697
Fatigue	VAS fatigue	r_s_ = −0.31	**0.002**
Depression and anxiety	HADS-total	r_s_ = −0.34	**< 0.001**
Depression	HADS-D	r_s_ = −0.45	**< 0.001**
Anxiety	HADS-A	r_s_ = −0.12	0.238
Confidence after stroke	CaSM-total	r_s_ = 0.47	**< 0.001**
Self-confidence	CaSM-self confidence	r_s_ = 0.43	**< 0.001**
Positive attitude	CaSM-positive attitude	r_s_ = 0.32	**0.002**
Social confidence	CaSM-social confidence	r_s_ = 0.30	**0.003**
**ICF domain: activities**			
Functional mobility	TUG	r_s_ = −0.25	**0.016**
Locomotor performance	ABILOCO-CS	r_s_ = 0.38	**< 0.001**
Manual performance	ABILHAND-CS	r_s_ = 0.20	**0.049**
Global performance	ACTIVLIM-CS	r_s_ = 0.39	**< 0.001**
**ICF domain: activities & participation**			
Satisfaction in activities & participation	SATIS-Stroke	r_s_ = 0.70	**< 0.001**
**Quality of life**			
Health-related quality of life	EQ-5D-5L	r_s_ = 0.32	**0.001**

mRS, modified Rankin Scale; VAS, Visual Analogic Scale; HADS, Hospital Anxiety and Depression Scale; CASM, Confidence after Stroke Measure; TUG, Timed up & Go; EQ-5D-5L, EuroQol - 5 Dimensions - 5 Levels; r_s_, Spearman's correlation; U, Mann-Whitney Rank Sum Test; H, Kruskal-Wallis one-way ANOVA on Ranks. *p*-values < 0.05 are highlighted in bold.

In contrast, sex, time since stroke, pain, and anxiety were not significantly related to PILS-Stroke social participation measures. No significant associations were found with age or stroke type; however, the data indicated a tendency toward reduced social involvement with increasing age as well as slightly greater social involvement following ischemic as compared to hemorrhagic stroke.

## 4 Discussion

The purpose of this study was to develop the PILS-Stroke questionnaire, a new instrument specifically designed to measure social participation through the perspective of involvement in meaningful life situations in adults living with long-term consequences of stroke. Following the Rasch procedure, the final version of the questionnaire comprises 38 relevant, unidimensional, and locally dependent items showing well-discriminated response categories with a common rating, and an invariant item difficulty hierarchy across age, sex, degree of disability, time since stroke, and risk of anxiety/depression.

### 4.1 Scale content and reliability

PILS-Stroke is the first European tool specifically designed to measure post-stroke social involvement across all ICF (7) participation subdomains. It demonstrated satisfactory internal consistency, with a PSI of 0.89, although this value was slightly lower than that reported for some other Rasch-built participation-related measures (16, 38). This may be partly explained by slight mistargeting of the scale to the mean social involvement level of the participants. Notably, the hierarchy of item difficulty aligns with previous findings, suggesting that situations requiring greater physical demands are perceived as being more difficult (16, 30). While the reliability of the instrument might potentially be improved by including more challenging items, such items (e.g., “Contribution to society”) are often less universal and therefore less relevant for many individuals (8). Furthermore, the high overall social involvement level of individuals in our sample may have contributed to mistargeting. Despite these reservations, the PSI was sufficient to distinguish four distinct levels of social involvement, thus supporting the tool's clinical and research utility (39).

### 4.2 Construct validity

The relationships between PILS-stroke measures and sociodemographic/clinical parameters are not only relevant for scale construct validation, but are also a matter of clinical interest. Indeed, previous work of this type (37) has generally relied on measures that assess participation in terms of frequency or satisfaction (but not involvement), and that combine the ICF activity and participation concepts (19). In contrast, the PILS-Stroke questionnaire aims specifically to capture the patients' sense of involvement in life situations requiring social interactions or relating to social roles.

#### 4.2.1 Sociodemographic contextual factors

Age tended to show an inverse relationship with social involvement in our sample. This trend may reflect the greater severity of stroke-related impairments and the higher prevalence of comorbidities observed in older populations, both of which usually adversely impact participation (37, 40). We did not detect any sex-related disparities in PILS-Stoke measures. Although prior studies have suggested that women may experience lower participation levels after a stroke (37), findings in the literature are inconsistent as no sex differences has also been reported (16).

#### 4.2.2 Clinical contextual factors

Participants with an ischemic lesion tend to be slightly more involved in life situations than those with a hemorrhagic lesion. To date, there is no consensus in literature regarding the influence of stroke type on participation There are reports of better functional outcomes for hemorrhagic stroke (41), while others claim the opposite (42). The time since stroke onset showed no meaningful correlation with reported social participation, aligning with recent findings suggesting that the prevalence of participation restrictions tends to persist over time following a stroke (3, 43). Finally, there was a positive correlation between decreased social involvement with the severity of disability (assessed with the mRS), as expected based on previously published literature (37, 44).

#### 4.2.3 Clinical measures

Most clinical assessments (measuring body functions, activities, activities & participation, and quality of life) used in this study demonstrated significant correlations with the level of social involvement in meaningful life situations, as measured by the PILS-Stroke questionnaire.

In the domain of **body functions**, there was a moderate correlation between depressive symptoms and social participation, aligning with previous studies (37, 44–46). Depression affects about one in four stroke survivors (3) and is known to limit participation and to be exacerbated by social isolation (47). Addressing depression may therefore be an important clinical target to improve social participation. In contrast, anxiety showed no significant association with social involvement in our study, although prior research has linked it to depression, reduced quality of life (48) and poorer daily functioning (49). All three subdomains of post-stroke confidence (i.e., self-confidence, positive attitude, and social confidence) showed significant correlations with social participation, ranging from weak to moderate, and with the strongest association observed for self-confidence. This aligns with prior literature indicating that self-related outcomes and positive attitudes facilitate social participation, while negative perceptions from others can act as barriers to social reintegration (50). In addition, our findings corroborate the Family of Participation-Related Constructs (fPRC) framework (9), which explicitly identified sense of self, including confidence, satisfaction, self-determination, and self-esteem, as interacting reciprocally with participation.

Within the **activity** domain of the ICF framework, social participation correlated more strongly with locomotor performance than with manual abilities. This finding is supported by the hierarchy of item difficulty (Figure 1), wherein life situations that demand physically active locomotion to move around the community appear among the most challenging items. Interestingly, functional mobility showed only a weak correlation with social participation, confirming that involvement in life situations seems more related to locomotor performance in real-life contexts (as measured by ABILOCO-CS) than to standardized measures of locomotor capacity (e.g. TUG) (7). A moderate correlation was found with global activity performance, reinforcing the prior evidence supporting a closer relationship between activity and social participation domains (37, 50). However, individuals reporting no limitation in global activity performance (i.e., achieving 100 ACTIVLIM-CS units) still exhibited a wide range of scores (extending from 50 to 100 PILS units) in their social participation measures (Figure 2D). There was a similar pattern at lower levels of activity performance, where variability in social participation remained substantial. This finding underscores the conceptual and empirical distinction between the activity and participation domains within the ICF (7) and fPRC (9) frameworks, and highlights the importance of assessing each domain independently to capture the full complexity of post-stroke functioning.

The strong association observed between **satisfaction regarding involvement in activities & participation** (SATIS-Stroke) and scores obtained at PILS-Stroke supports its convergent validity, as both scales are notably measuring related yet distinct constructs (i.e., satisfaction and social involvement) within the participation domain. Finally, the highlighted significant but weak correlation between social participation measures and health-related **quality of life** confirms the established finding that participation is one of the multiple predictors of quality of life in elderly patients with physical disabilities (51).

### 4.3 Environmental contextual factors that may influence social participation

In our analysis of convergent validity, we focused exclusively on the relationships between social participation and personal factors, without examining environmental and cultural determinants. However, there is growing evidence that environmental factors substantially influence post–stroke participation. Environmental features such as environmental adaptations facilitating independence, accessibility of services, transportation, physical surroundings, social support and the attitudes of others have consistently been identified as major facilitators or barriers of participation (52, 53). Moreover, cultural values influence social participation–related behaviors, shaping individuals' expectations, social norms, and the perceived legitimacy of requesting help (53). A systematic review further indicates that, although socio–demographic determinants show inconsistent relationships with participation, psychosocial factors (e.g., motivation, self–esteem, acceptance) and environmental factors (e.g., support networks, social attitudes, physical environment, and access to healthcare and caregiving services) are recurrent determinants across studies (36). Additionally, a study using structural equation model analysis highlighted that social attitudes and physical environment appear to mediate the indirect influence of systems, services, and policies on participation (54). Altogether, these findings demonstrate the interplay between personal, environmental, and cultural factors in shaping social participation, highlighting the need for future validation studies using PILS–Stroke to integrate environmental and cultural determinants as part of the social participation framework.

### 4.4 Strengths, limitations, and perspectives

To the best of our knowledge, the PILS-Stroke is the first Rasch-built questionnaire designed to measure participation through the lens of social involvement among individuals living with the consequences of stroke within a European setting. Its high measurement precision, strong clinical relevance, and ease of use, combined with free online access via https://www.rehab-scales.org, provide new opportunities for both clinical practice and research applications.

We note several limitations of the present study. First, although the sample size of 100 patients is generally accepted to be sufficient for the calibration of a scale for most purposes (21), the slight mistargeting observed may have reduce the accuracy of parameter estimates. In fact, inclusion of more assessments of less involved patients might have enhanced the scale precision. However, such patients are inherently less likely to take part in research. Moreover, a larger sample size would be required to formally investigate scale invariance. Although the current sample appears sufficient to identify life situations exhibiting major differential item functioning (DIF) across clinically relevant subgroups, the presence of minor DIF effects cannot be excluded. According to Scott et al. (55), at least 100 and preferably 200 respondents per group are needed to detect more subtle DIF. Further research with a larger sample could therefore be warranted to more comprehensively examine the invariance of PILS-Stroke.

Second, we concede that the experimental 72-item version of PILS-Stroke was time-consuming and sometimes tiring, in addition to the other assessments. Although PILS-Stroke was always conducted at the beginning of the experimentation, fatigue may have had a slight effect on social participation measures. Despite its relatively modest final length of 38 items, the final scale's consequential validity warrants further evaluation. If respondent fatigue emerges as a definite concern, it would be important in future research to consider developing a shortened form or to explore a computerized adaptive testing (CAT) approach.

Third, we note that cognitive screening was not consistent throughout the entire study period. The Mini-Mental State Examination (MMSE) was used for the first 12 participants, after which we adopted the Montreal Cognitive Assessment (MoCA), once the assessors completed the required certification. Although both tools assess cognitive impairment using a 30-point scale and include overlapping domains, differences in sensitivity and item structure may affect the comparability of cognitive scores. However, a common cut-off (< 10/30) is reported in the literature to indicate severe cognitive impairment (56–58). We took this deliberately permissive threshold to exclude only those individuals whose cognitive impairment would have precluded meaningful self-report. Importantly, some cognitive functions assessed by the MMSE or MoCA (e.g., naming, visuospatial abilities, or orientation) may be affected by stroke-related lesions, but without being essential for questionnaire completion, which may explain why mild to moderate cognitive impairment did not appear to compromise response patterns, as supported by satisfactory person-fit statistics.

Fourth, the sample mainly included community-dwelling survivors, reflecting the original intent of PILS-Stroke to capture social involvement across a broad range of life situations. This limits the generalizability of our findings (such as relationships observed between PILS-Stroke and clinical measures) to institutionalized individuals, whose participation occurs within a more restricted and context-specific environment (e.g., participation in care, leisure limited to those offered by the institution). In addition, individuals who were highly isolated or severely impaired were only minimally represented. Our findings should therefore be considered representative only for community-dwelling populations with slight to moderate disability. Further investigations are needed to verify the usability of the scale in these populations.

Finally, the study relied on a single dataset and did not include an external validation sample, increasing the potential risk of sample dependent item calibration. This risk is mitigated using the Rasch model thanks to its specific objectivity property (33, 59) implying that, when model assumptions are met (good fit statistics), subjects' measures are in principle independent of the relative difficulty of the particular set of items used, and similarly, relative item difficulty estimates are independent of the particular sample used for calibration. Future research should, however, include cross-validation in a more heterogeneous sample to confirm the stability of item difficulty estimates, improve targeting across the full continuum of social participation levels, and further support the generalizability of the measurement structure beyond the present dataset. The validation of an instrument is a continuous process, and no single study can comprehensively establish all its psychometric properties. In addition to cross-validation, future studies are needed to assess test-retest reliability and sensitivity to change of PILS-stroke to ensure its ability to capture clinically significant changes in social participation among stroke patients.

## 5 Conclusion

PILS-Stroke provides a direct and clinically relevant measurement to assess perceived social involvement in meaningful life situations, a major outcome and therapeutic goal in both research and clinical practice among individuals living with post-stroke disabilities. It addresses a gap in the availability of psychometrically sound instruments assessing this core dimension of social participation within European populations. Future research should focus on evaluating its responsiveness to changes and test-retest reliability over time, as well as its applicability across diverse populations, particularly among highly isolated or severely impaired individuals.

## Data Availability

The raw data supporting the conclusions of this article will be made available by the authors, without undue reservation.
