# Supplementary material for: “How involved do you feel?” The PILS-Stroke questionnaire: a Rasch-built measure of social participation after stroke
Source: Front Neurol. 2026 May 11;17:1733609. doi: 10.3389/fneur.2026.1733609 (PMC13199013; doi:10.3389/fneur.2026.1733609)
Supplement: Supplementary file 1 [file Table_1.docx]

Supplementary Material

# Supplementary Methods

## PILS-Stroke instructions

**Purpose:**

PILS-Stroke is a self-report questionnaire for people living with post-stroke disabilities that measure social participation.

**Important considerations:**

- The questionnaire must preferably be completed by patients themselves (alone).
- Examinators should never influence patients’ answers.
- If patients have motor/visual impairments or mild to moderate executive functions’ deficits (that can be assessed with MoCA/MMSE), examinators are allowed to conduct the questionnaire throughout an interview. In this case, they may read items **without rephrasing questions or influencing answers** and/or transcribe responses.
- PILS-Stroke is **not appropriate** for people with severe cognitive impairment.

**How to complete the questionnaire:**

1. Understanding the topic of the questionnaire

Patients are asked to rate their **level of involvement** in several life situations. Examinators should first read the two following definitions:

**Social participation:** the involvement of people in life situations, chosen by individuals because they make sense to them, in which they may interact with others, or which relate to social roles.

**Involvement**: feeling engaged and paying attention to the situation one is in through interactions and with a certain level of motivation and commitment (being attentive, giving your opinion, encouraging, doing or helping to do tasks).

1. Understanding how to respond to the questionnaire

For each item, patients must, at first, choose whether they get involved in the situation or not.

- If they do not, they must choose between "I don't want to/I don't know" if they are not interested in or concerned by the situation or "I would like to but I don't get involved".
- If they are involved in the situation patients must choose between "I get slightly involved" or "I get very involved".

The following description of **each rating choice must be read to patients** before completing the PILS-Stroke questionnaire:

**• "I don't want to/I don't know":**

You are not involved in this life situation because you do not want to be, or you do not have to. You choose not to participate because it is not something you want to be able to do. You can also select this answer if you do not know what to say because you do not remember or have never experienced the situation.

**• "I would like to but I don't get involved":**

When you encounter this life situation, you do not get involved because you do not have the opportunity even though you would like to. Choose this response if you are not involved in something you wish you could do or are supposed to do.

**• "I get slightly involved":**

When you encounter this life situation, you get involved at least a little bit, even if you need help. Choose this response if you do not feel as involved as you would like to be but can still participate to some extent.

**• “I get very involved”:**

When you encounter this life situation, you get very involved, no matter how much help you need. You feel fully included and are participating in (almost) everything. "

Finally, **an example should be provided** to help to distinguish the two choices where the patient does not get involved:

*“Let’s say that, since you had a stroke, you do not participate in cultural events. If you don’t because you are not interested in it (so as before stroke occurs or since then), choose "I don't want to/I don't know". In contrast, if you don’t participate in sport events since your stroke, but you still have interest in it and feel restricted, then choose "I would like to but I don't get involved”. Also, if you are not concerned by the situation (for example, having no siblings while the item is related to siblings), you must also choose "I don't want to/I don't know".*

## Development of the experimental version of PILS-Stroke

Ninety-six potentially relevant items were identified and selected from seven participation assessment tools in the literature: Participation Measurement Scale (PM-scale)^1^, Reintegration to Normal Living Index (RNLI)^2^, Participation Enfranchisement^3^, Community Participation Activation Scale (CPAS)^4^, SATIS-Stroke^5^, the Indian Stroke Scale (ISS)^6^ and LIFE-H^7^. These items were translated and adapted as necessary (e.g., converting interrogative items into affirmative statements; adding some elements to ensure that the items relate to interactions with others or social roles). In addition, 35 additional items were devised to provide a comprehensive coverage of all subcategories within the participation domain of the ICF^8^. Three experts (i.e., physiotherapists and researchers) were asked to review and adapt/remove items if they did not align with the chosen definition of participation (especially the notion of involvement rather than attendance and the distinction with the activity domain of the ICF), the format of the PILS-Stroke questionnaire (i.e., affirmative sentences). After a thorough analysis of the item content by the three experts, 44 items were removed.

To ensure scale content validity, this item pool (n=87) was submitted to five physiotherapists and five stroke survivors for at least six months to assess the clarity of the items and their relevance considering the participation definition and the European sociocultural context. Items were retained if they were deemed relevant by at least 60% of patients and 80% of therapists. However, seven clinically relevant items (unanimously endorsed by all therapists), although not reaching the patients’ endorsement threshold, were retained as patients’ responses might have been influenced by their sociodemographic characteristics (all were males and aged ≥60 years). For example, the item “Cleaning my house to welcome guests” may not have been perceived as relevant by these patients, as this life situation is still more often socially ascribed to women. Following patients’ and therapists’ review, 15 irrelevant items were removed, leading to a 72-item experimental version of the PILS-Stroke questionnaire that has been submitted to participants.

## Detailed Rasch analysis procedure

1. **Response category discrimination.** Participants were asked to report their level of involvement on a 3-level scale: “I would like to, but I don’t get involved” (0), “I get slightly involved” (1), “I get very involved” (2). When thresholds were well ordered, categories were assumed well discriminated. In contrast, if thresholds were disordered, it meant that at least one response category was not discriminated. When this expected pattern was not observed for an item as highlighted by disordered thresholds between response categories, such item was then discarded.
2. **Similar relative thresholds (relative to item difficulty).** The use of the RSM implies to select only items with similar distances between thresholds locations. Items with relative threshold locations significantly differ­ent from the average (Z-scores out of ±2) were removed.
3. **Unidimensionality.** Each item must measure the same unique underlying trait (in our case, subjects’ social involvement); this is known as unidimensionality. Various individual item fit statistics^9^ were used to verify that the observed data fit the responses predicted by the Rasch model under the assumption of unidimensionality. Only items presenting Fit Residuals (FitRes) between -2.5 and +2.5 and a non significant (i.e., p>0.05) Chi-square (Chi²) test probability met the requirements for unidimensionality and were retained. The FitRes was calculated as the difference between observed and expected scores divided by the standard deviation of the expected score for each of the 3 class intervals then normalized to a standardized normal distribution; the Chi² was computed as the sum of squares of the standardized residual for each class interval of increasing involvement levels, and a global Chi^2^ for the entire final item pool was also calculated to verify that all items contribute to the definition of a unidimensional participation measure. A no significant global fit indicator indicated an overall satisfactory scale unidimensionality.
4. **Scale invariance.** Differential Item Functioning (DIF) analyses were conducted to ensure that the items present invariant item difficulty hierarchy across age (≤ 63 years [median age] vs. > 63 years), gender (males vs. females), level of disability (mRS 0–2 [no or slight disability] vs. mRS 3–5 [moderate to severe disability]), time since stroke (≤ 37 months [median time] vs. > 37 months), and risk of anxiety/depression (Hospital Anxiety and Depression Scale (HADS) 30 ≤ 12 [median score] vs. > 12). DIFs were investigated through a two-way analysis of variance on the standardized residuals of 3 interval classes of increasing involvement levels. Items with a significant main effect on patients’ characteristics (age, gender, level of disability, time since stroke and risk of anxiety/depression) showed uniform DIF and were discarded.
5. **Local independence**. Items are expected to exhibit local independence, meaning that responses to one item should not be dependent on responses to another, after accounting for the latent trait^10^. This criterion is assessed by examining the residual correlation matrix. One item from each pair of items showing moderate to strong residual correlations (i.e., ≥ 0.4) were removed to maintain local independence^11, 12^.

# Supplementary Results

## Supplementary Tables

| Table S1. Median and range values of clinical measures (body functions, activities, activities & participation, and quality of life). | | | |
| --- | --- | --- | --- |
|  |  | **Median (Q1-Q3)** | **Range** |
| *ICF domain: body functions* | | | |
| Pain | VAS pain | 3 (1-5) | 0-8 |
| Fatigue | VAS fatigue | 5 (3-6) | 0-10 |
| Depression and anxiety | HADS-total | 12 (9-16) | 3-31 |
| Depression | HADS-D | 6 (3-8) | 0-16 |
| Anxiety | HADS-A | 6 (4.25-9) | 1-18 |
| Confidence after stroke | CaSM-total | 48 (42.5-54) | 17-76 |
| Self-confidence | CaSM-self confidence | 12 (9-15) | 4-25 |
| Positive attitude | CaSM-positive attitude | 15 (13-17) | 1-24 |
| Social confidence | CaSM-social confidence | 20 (17-24.5) | 5-30 |
| *ICF domain: activities* | | | |
| Functional mobility | TUG (s) | 13.9 (10.8-17.7) | 6.34-107 |
| Locomotor performance | ABILOCO-CS | 81.9 (70.8-100) | 0-100 |
| Manual performance | ABILHAND-CS | 60.4 (50.0-76.6) | 30.1-100 |
| Global performance | ACTIVLIM-CS | 66.2 (58.1-72.9) | 32.6-100 |
| *ICF domain: activities & participation* | | | |
| Satisfaction with activities/participation | SATIS-Stroke | 58.3 (54.8-66.3) | 46.7-100 |
| *Quality of life* | | | |
| Health-related quality of life | EQ-5D-5L | 0.72 (0.51-0.83) | -0.078-1 |

**Table S2. Items deleted from the experimental version of PILS-Stroke.**

| Delete reason | Item statement |
| --- | --- |
| Too low frequency | Protect yourself when having sexual relationships |
| Thresholds disorder | Organize transportation to get me where I need to go |
| Thresholds disorder | Go to the hospital for care |
| Thresholds disorder | Move around the house to participate in family life (all rooms, all floors) |
| Thresholds disorder | Use a car (as driver or passenger) to go somewhere (visit a loved one, run errands...) |
| Thresholds disorder | Take care of a pet |
| Thresholds disorder | Do the job/volunteer work that I like |
| Thresholds disorder | Go sightseeing in my country (or a neighboring country) |
| Thresholds disorder | Travel to distant countries |
| Thresholds disorder | Practice a sport (tennis, fitness, Nordic walking, soccer...) |
| 2≤Z-score ≤ -2 | Stay informed about current events |
| 2≤Z-score ≤ -2 | Enter and leave my home to meet my neighbors |
| 2≤Z-score ≤ -2 | Be mindful of my appearance before meeting people |
| 2≤Z-score ≤ -2 | Use restrooms outside my home (public toilets, pool showers, sinks...) |
| Local dependence | Participate in the care of my children and grandchildren |
| Local dependence | Do the hobbies I enjoy |
| Multidimensionality | Try to make others laugh (show my sense of humor) |
| Multidimensionality | Understand other people’s jokes and sarcasm |
| Multidimensionality | Choose clothes that fit the occasion |
| Multidimensionality | Get involved in the decision-making of those around me |
| DIF gender | Share my knowledge within a group (family, friends, coworkers...) |
| DIF gender | Express my gratitude to someone |
| DIF gender | Have a fulfilling emotional life |
| DIF gender/mRS | Go out at night or in the dark to attend an evening event |
| DIF mRS | Move around in the city to go shopping or attend an event even when there are obstacles (construction, blocked sidewalks...) |
| DIF mRS/Age | Change clothes during the day if necessary for my social activities (sports, parties, work...) |
| DIF mRS/Age | Set the table when I have guests |
| DIF Age | Share my emotions |
| DIF Age | Send an email to someone I don't know personally |
| DIF Age | Clean my house to receive guests |
| DIF Age/HADS | Discuss something I read with someone |
| DIF HADS | Vote in elections |
| DIF Time since stroke | Attend artistic and cultural activities (movies, theater, ...) |
| DIF Time since stroke | Participate in ceremonies (weddings, birthdays, etc.) |

# Supplementary Discussion

## Structural validity

After removing items whose response categories were not well discriminated or did not share the same rating scale, the unidimensionality requirement was investigated and only items with observed data fitting the responses predicted by the Rasch model were retained. This step was essential to ensure that PILS-Stroke captures a single latent construct, namely perceived involvement in meaningful life situations following stroke. For example, items such as “Try to make others laugh (show my sense of humor)” and “Understand other people’s jokes and sarcasm” were excluded due to significant misfit, likely because they refer to additional dimensions such as personality traits or social cognition, rather than core aspects of participation.

Sequential DIF analysis led to the removal of items showing differential functioning across variables (e.g., older participants more likely to endorse “share my emotions,” younger ones “send an email to someone I don’t know”). Such differences may compromise scale invariance as items’ difficulty is perceived differently across different subgroups. However, to balance psychometric robustness with clinical relevance, one item with marginal DIF (i.e., “Call someone I know by phone or cell phone,” difficulty: 53.9 PILS units) was retained for its meaningful content.

**
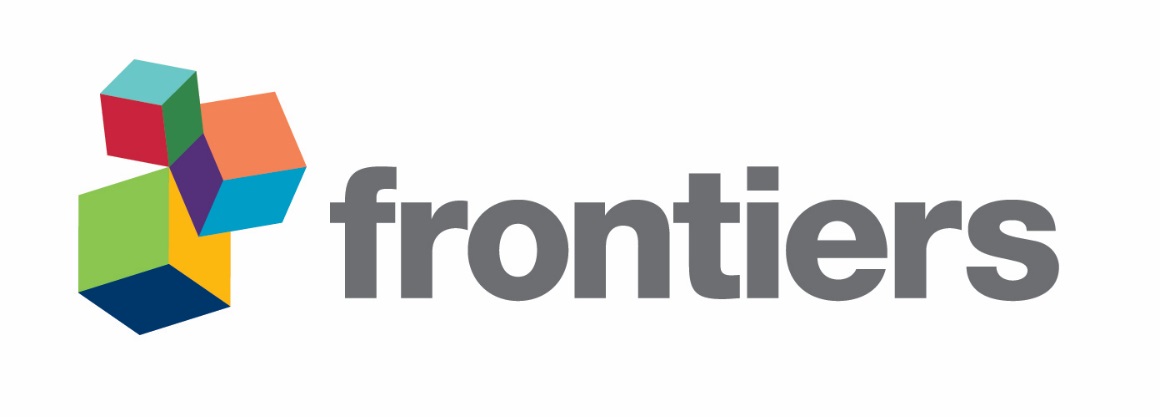
**

References

1. Kossi O, Nindorera F, Batcho CS, Adoukonou T, Penta M, Thonnard JL. Measuring Participation After Stroke in Africa: Development of the Participation Measurement Scale. Arch Phys Med Rehabil. 2018;99(4):652-9.

2. Wood-Dauphinee SL, Opzoomer MA, Williams JI, Marchand B, Spitzer WO. Assessment of global function: The Reintegration to Normal Living Index. Arch Phys Med Rehabil. 1988;69(8):583-90.

3. Heinemann AW, Lai JS, Magasi S, Hammel J, Corrigan JD, Bogner JA, et al. Measuring participation enfranchisement. Arch Phys Med Rehabil. 2011;92(4):564-71.

4. Lee D, Hammel J. Development of the Participation Activation Scale. Archives of Physical Medicine and Rehabilitation. 2016;97(10):e79.

5. Bouffioulx E, Arnould C, Thonnard JL. SATIS-Stroke: A satisfaction measure of activities and participation in the actual environment experienced by patients with chronic stroke. J Rehabil Med. 2008;40(10):836-43.

6. Prakash V, Ganesan M. The Indian Stroke Scale: Development and validation of a scale to measure participation in daily activities among patients with stroke in India. Int J Stroke. 2021;16(7):840-8.

7. Desrosiers J, Noreau L, Robichaud L, Fougeyrollas P, Rochette A, Viscogliosi C. Validity of the Assessment of Life Habits in older adults. J Rehabil Med. 2004;36(4):177-82.

8. World Health O. International classification of functioning, disability and health : ICF. Geneva: World Health Organization; 2001.

9. Smith EV, Jr. Detecting and evaluating the impact of multidimensionality using item fit statistics and principal component analysis of residuals. J Appl Meas. 2002;3(2):205-31.

10. Yen WM. Effects of Local Item Dependence on the Fit and Equating Performance of the Three-Parameter Logistic Model. Applied Psychological Measurement. 1984;8(2):125-45.

11. Sogbossi ES, Loutou A, Noukimi DA, Ahouandjinou SM, Houssou A, Bleyenheuft Y, et al. Cross-cultural adaptation of the West and Central African version of the ABILHAND-Kids questionnaire for children with cerebral palsy. Disabil Rehabil. 2025:1-13.

12. Vaccarino AL, Black SE, Gilbert Evans S, Frey BN, Javadi M, Kennedy SH, et al. Rasch analyses of the Quick Inventory of Depressive Symptomatology Self-Report in neurodegenerative and major depressive disorders. Front Psychiatry. 2023;14:1154519.
